# Sex Differences and Role of Gonadal Hormones on Glutamate LevelAfter Spinal Cord Injury in Rats: A Microdialysis Study

**DOI:** 10.32598/bcn.9.10.260

**Published:** 2019-05-01

**Authors:** Razieh Samandari, Majid Hassanpour-Ezatti, Sajad Fakhri, Fatemeh Abbaszadeh, Masoumeh Jorjani

**Affiliations:** 1. Department of Physiology, Faculty of Basic Sciences, Shahed University, Tehran, Iran.; 2. Pharmaceutical Sciences Research Center, Health Institute, Kermanshah University of Medical Sciences, Kermanshah, Iran.; 3. Neuroscience Research Center, Shahid Beheshti University of Medical Sciences, Tehran, Iran.; 4. Department of Pharmacology, School of Medicine, Shahid Beheshti University of Medical Sciences, Tehran, Iran.

**Keywords:** Spinal Cord Injury, Microdialysis, Glutamate, Sex hormones

## Abstract

**Introduction:**

Sex differences in outcomes of Spinal Cord Injury (SCI) suggest a sex-hormone-mediated effect on post-SCI pathological events, including glutamate excitotoxicity. This study aimed to investigate the importance of gonadal hormones on glutamate release subsequent to SCI in rats.

**Methods:**

After laminectomy at T8–T9, an electrolytic lesion was applied to the spinothalamic tracts of male and female rats. Using spinal microdialysis, we assessed glutamate levels at the site of lesion in both intact and gonadectomized rats for 4 hours. In this way, we examined the sex differences in the glutamate concentrations.

**Results:**

The peak retention time of glutamate level was 10.6 min and spinal glutamate concentration reached a maximum level 40 min following SCI. In male SCI rats, gonadectomy caused a significant elevation of glutamate level (P<0.001) following injury which was maximum 40 min post-SCI as well. However, no significant alterations were seen in gonadectomized female rats.

**Conclusion:**

The significant differences in glutamate levels between both intact and gonadectomized SCI male and female rats show the sex-hormone-related mechanisms underlying the molecular events in the second phase of SCI. It seems that the role of male gonadal hormones to prevent glutamate excitotoxicity is more prominent. The exact mechanisms of these hormones on the functional recovery after SCI should be clarified in further studies.

## Highlights

- There is a sex difference in the extracellular glutamate level in the spine of rats.
- The maximum concentration of glutamate is detected 40 minutes after Spinal Cord Injury (SCI).
- Gonadectomy does not significantly change the spinal glutamate levels in female or male rats.
- SCI-induced glutamate release increases with the gonadectomy of male animals.

## Plain Language Summary

Medical complications after Spinal Cord Injury (SCI) are common and serious in most patients. Glutamate is an excitatory amino acid which plays an important role in the development of post-SCI complications. There is also some evidence on the gender-related differences in response to spinal injury. To examine the contribution of gonadal hormones on sex differences in post-SCI events, we measured the glutamate level following SCI in intact and gonadectomized rats. Based on the results, a significant decrease in gonadal hormones after castration in male rats was seen that led to a noticeable increase in the spinal glutamate level after SCI. This evidence suggests the benefits of male gonadal hormones in the reduction of glutamate excitotoxicity, in the context of SCI.

## 1. Introduction

Spinal Cord Injury (SCI) is a disturbing incident (Naghdi et al., 2017; Silva, Sousa, Reis, & Salgado, 2014; Sun et al., 2016) associated with primary and secondary pathophysiological events, and affects the activity of several neurotransmitters like glutamate. After primary trauma to the cord, neurons in the spinal cord continue to die for hours (Cho, Hachem, & Fehlings, 2018; İsmailoğlu, Oral, Görgülü, Sütçü, & Demir, 2010; Liu, Thangnipon, & McAdoo, 1991; Morin, 2018). The release of harmful substances in the Central Nervous System (CNS) upon trauma is widely held responsible for the secondary damages to adjacent neural tissue (Gaudet, Popovich, & Ramer, 2011; Lian, Lu, Chang, & Zhang, 2018; Liu et al., 1991; Liu, Xu, Pan, & McAdoo, 1999).

The amino acid glutamate, as the major Excitatory Amino Acid (EAA) in the CNS (Benton, Ross, & Miller, 2000; Doolen, Blake, Smith, & Taylor, 2012; Lian et al., 2018; Weiss, Doshi, Sinha, Liu, & Chi, 2002), significantly increases after SCI (Hulsebosch, Hains, Crown, & Carlton, 2009; Smith, Somogyi, Bird, Chancellor, & Boone, 2002). Extracellular glutamate in high concentration binds to α-Amino-3-hydroxy-5-Methylisoxazole-4-Propionic Acid, kainate (AMPA), and N-Methyl-D-Aspartate (NMDA) receptors, results in intracellular elevation of calcium level. As a result, neuronal cells die due to the activation of proteases and endonucleases and also generation of free radicals (Maiolino, Lariccia, Amoroso, Angelova, & Abramov, 2017; Smith et al., 2002; Sribnick, Del Re, Ray, Woodward, & Banik, 2009; Van Laar et al., 2015).

Although glutamate; as the main cause of neuronal cell death after SCI, Traumatic Brain Injury (TBI), and stroke (Barkhoudarian, Hovda, & Giza, 2011; Sribnick et al., 2009); plays an important role in pathophysiologies of neurodegenerative diseases and normal function of CNS, the sex differences affecting multiple neurotransmitter systems and functional recovery mediated by gonadal hormones (Ribeiro, Vieira, Pires, Olmo, & Ferguson, 2017; Wagner et al., 2007) are of special importance. In the present study, the role of sex differences and gonadal hormones on the glutamate release after SCI is investigated.

## 2. Methods

### 2.1. Animal preparation and microdialysis fiber insertion

Sprague-Dawley male and female rats (weight: 220–270 g) were kept in standard cages (4–6 rats per cage) in a 12 h:h light/dark temperature-controlled room (24±1°C). The animals received water and food ad libitum. All the experiments were performed in compliance with the National Institutes of Health Guide for the Laboratory Animals Use and Care and approved by the Ethics and Research Committee of Shahid Beheshti University of Medical Sciences. The rats with both sexes were divided into three groups; GDX (gonadectomized), SCI (received spinothalamic tract lesioning), and GDX+SCI (received spinothalamic tract lesioning two weeks after gonadectomy).

The animals were anesthetized with a mixture of ketamine (80 mg/kg IP) and xylazine (10 mg/kg IP) for microdialysis experiments. Their dorsal parts were shaved and washed with Betadine as an antiseptic. The spinal cord was exposed by removing the bone and muscle at T8–T9 level (Xu, Hughes, Ye, Hulsebosch, & McAdoo, 2004).

The microdialysis fiber was prepared by joining the silica tube to a semipermeable membrane so that a 2-mm dialysis zone could be observed between the silica tubes. The fibers were inserted laterally through the cord at segments T8–T9 exactly at the site of the lesions. Artificial cerebrospinal fluid (ACSF; all in mM: 26 NaHCO_3_, 2 MgSO_4_, 1.25 NaH_2_PO_4_, 114 NaCl, 1 CaCl_2_, 10 glucose, 1 NaOH, and 3 KCl) was pumped through the fiber at a rate of 2 μL/min. The sample collection was started 75 to 90 min after inserting the fibers into the cord (Liu et al., 1991). The delay was intended to permit the depletion and release of amino acids induced by fiber insertion to be stabilized (Xu, McAdoo, Hughes, Robak, & De Castro, 1998). Continuous collection of the fluid was done from the outlet end of the microdialysis fibers in ice in plastic tubes (Liu et al., 1991). After 40 min of sample collection, the spinal cord was injured in the zone around the microdialysis fiber by an electrical lesion in the spinothalamic tract (Liu & McAdoo, 1993).

### 2.2. Spinothalamic tract lesion

The animals were anesthetized and underwent laminectomy. After dialysis, the right spinothalamic pathway was lesioned using a tungsten microelectrode placed 0.6–0.8 mm lateral to midline and 1.6–1.8 mm deep. A short-term current pulse (+300 μA, 90 s) was passed through the electrode and a ground electrode was placed in the muscle beside the spine (Wang & Thompson, 2008).

### 2.3. Operation

The male and female rats were deeply anesthetized with an intraperitoneal (IP) mixture of xylazine/ketamine (10/80 mg/kg) (McAdoo et al., 2005). They were then Gonadectomized (GDX) by removing their ovaries or testes. After two weeks of allowing animals to be recovered from the operation, these GDX rats were studied as a group (Mousavi, Shafaghi, Kobarfard, & Jorjani, 2007).

### 2.4. Glutamate detection

The fluid passing through the lumen of the probe was collected and analyzed for detection of amino acids, typically by High-Performance Liquid Chromatography (HPLC) with a fluorescence detector (Fakhri, Mohammadi, Jalili, Hajialyani, & Bahrami, 2018; Majnooni et al., 2014; Mohammadi, Tammari, Fakhri, & Bahrami, 2013) (excitation wavelength 340 nm, emission 475 nm cut-off filter) and a reverse-phase column (Shimpack CLC-ODS, 150 L×4.6 mm, 5 μm) (McAdoo & Wu, 2008).

**Figure 1.**
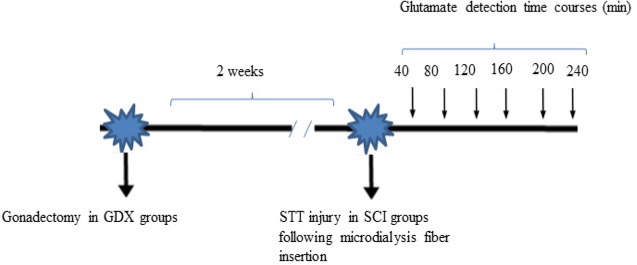
A summary of the study procedure GDX: Gonadectomized; SCI: Spinal Cord Injury; STT: Spinothalamic Tract

Amino acids were determined by forming an orthophthalaldehyde/2-mercaptoethanol derivative. The isocratic reverse-phase HPLC was used to determine the glutamate concentration (Lisi, Westlund, & Sluka, 2003; Zhu et al., 2011). The mobile phase consisted of a mixture of 100 mM disodium hydrogen phosphate solution, methanol, and acetonitrile (73:20:7 w/w/w) and then was adjusted to pH 6.1 with orthophosphoric acid (Smith et al., 2002) with the flow rate of 1 mL/min. Figure 1 displays the whole procedure of the current study.

### 2.5. Statistical analysis

The obtained results were expressed as Mean±SEM (standard error of the mean). Statistical analysis was conducted by repeated measurement of 1-way and 2-way Analysis of Variance (ANOVA) followed by Tukey and Bonferroni test as required. In all calculations, P<0.05 was regarded as the significance level.

## 3. Results

### 3.1. Influences of sex and gonadal hormones on baseline glutamate release

**Figure 2.**
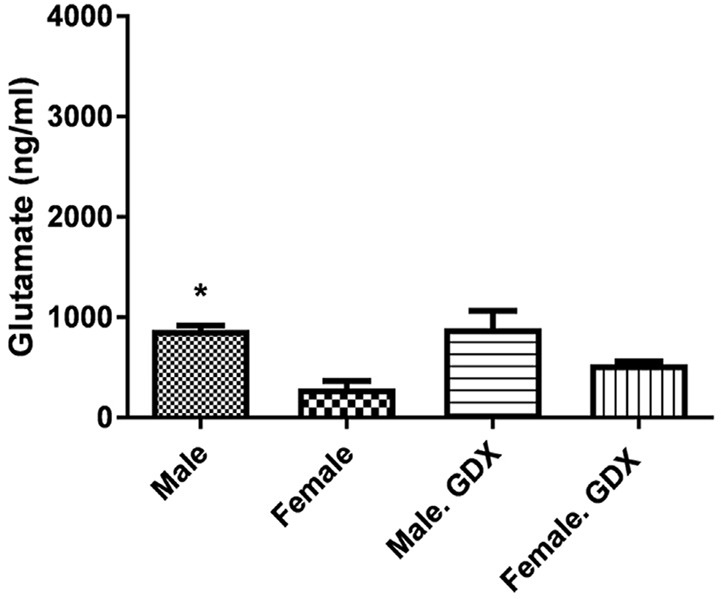
Sex differences in the spinal glutamate level in rats (n=3) Data are presented as Mean±SEM; The repeated measure of 1-way Analysis of Variance (ANOVA) followed by Tukey post hoc test. ^*^ P<0.05 intact male vs. female rats

The Mean±SEM baseline levels of glutamate were different between intact male (873.48±44.35 ng/mL) and female rats (290.24±76.19 ng/mL) with significant higher glutamate concentration in male rats (P<0.05) (Figure 2). Gonadectomy of animals did not significantly change the baseline glutamate levels in both sexes. Additionally, the pick corresponding to glutamate was appeared at 10.6 min.

### 3.2. Effect of SCI on the spinal glutamate level

**Figure 3.**
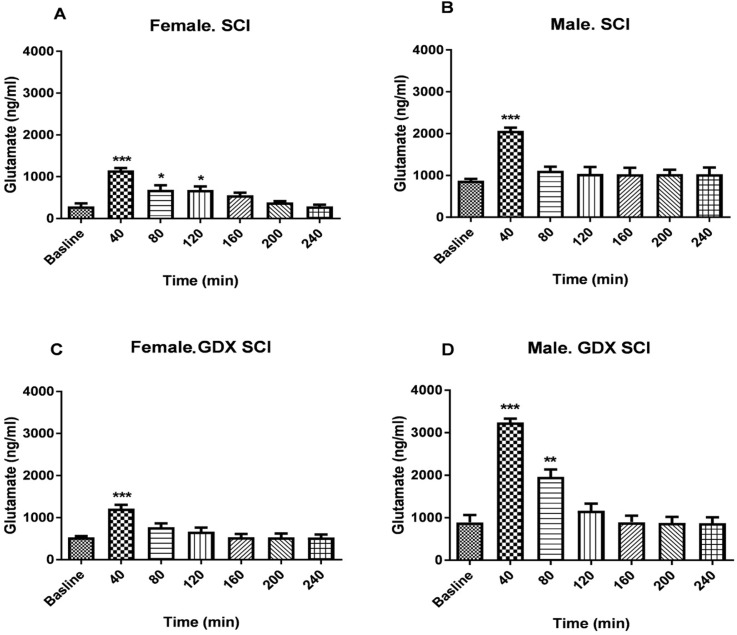
Changes in spinal glutamate level after Spinal Cord Injury (n=3) SCI female rats (A); SCI male rats (B); GDX+SCI female rats (C); and GDX+SCI male rats (D). The samples were collected during 40 min time and those started with a baseline and after injury continued for 4 h in both intact and gonadectomized rats. Data are presented as Mean±SEM. The repeated measure of 1-way Analysis of Variance (ANOVA) followed by Tukey test. ^*^ P<0.05; ^**^ P<0.01; ^***^ P<0.001 vs. baseline

**Figure 4.**
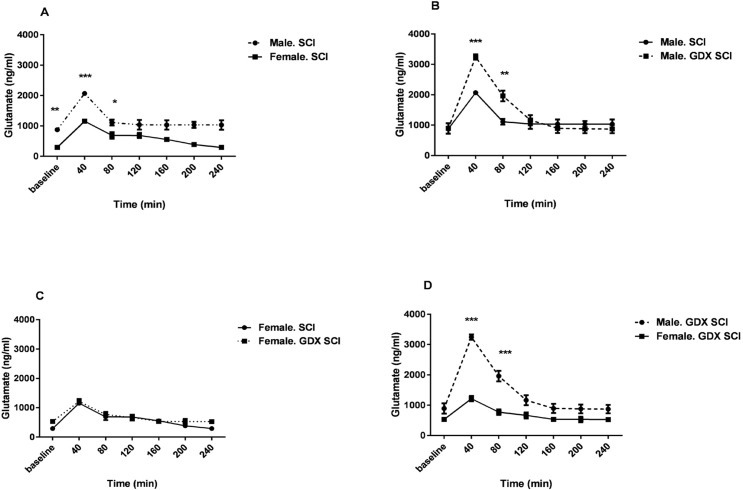
Influences of sex and gonadal hormones on the concentration of glutamate after Spinal Cord Injury (n=3) A. Male vs. female spinal cord injured rats; B. Male SCI rats vs. male GDX+SCI rats; C. Female SCI rats vs. female GDX+SCI rats; and D. Male GDX+SCI rats vs. female GDX+SCI rats. The samples were collected during 40 min time and those started with a baseline and after injury continued for 4 h in both intact and gonadectomized rats. Data are presented as Mean±SEM. Two-way Analysis of Variance (ANOVA) with Bonferroni post hoc test. ^*^ P<0.05; ^**^ P<0.01; ^***^ P<0.001 in A, B, C and D significantly different between two diagrams.

SCI produced a significantly greater glutamate level in all groups of rats after the injury (P<0.001) (Figure 3 and 4). The concentration of glutamate increased following the injury with a pick value at 40 min. The sharp increase occurred in the sample immediately after SCI. Figures 3 and 4 show that in the electrical injury model, glutamate release increased and then decreased rapidly and returned to near baseline within 80 min. After SCI, the Mean±SEM glutamate levels at 40 min following injury in both sexes (2068.11±76.49 in males and 1156.63±50.37 in females) was significantly greater than baseline (P<0.001) (Figure 3 A, B).

### 3.3. Effect of gonadectomy on spinal glutamate Level

Gonadectomy of rats did not change the Mean±SEM baseline glutamate levels in both male (873.48±44.35 ng/mL) and female rats (290.24±76.19 ng/mL). Glutamate levels changed in female GDX rats 40 min after SCI (P<0.001) (Figure 3 C). There were also significant differences in glutamate release 40 and 80 min after the injury in gonadectomized male animals (P<0.001) (Figure 3 D). Figure 4 A shows that male rats with SCI had a higher level of glutamate than female SCI rats 40 min (2068.11±76.49 ng/mL vs. 1156.63±50.37 ng/mL, P<0.001) and 80 min (1112.52±97.73 ng/mL vs. 687.63±110.84 ng/mL, P<0.05) after the injury.

Male GDX+SCI rats had a significantly higher Mean±SEM glutamate level than SCI male rats 40 min (3245.069±88.18 ng/mL vs. 2068.11±76.49 ng/mL, P<0.001) and 80 min (1960.57±173.53 vs. 1112.52±97.73 ng/mL, P<0.01) after the injury (Figure 4 B). However, this significant difference was not observed in female rats (Figure 4 C). Male GDX+SCI rats had also higher Mean±SEM glutamate level than female GDX+SCI rats 40 min (3245.069±88.18 ng/mL vs. 1215.81±88.65 ng/mL) and 80 min (1960.57±173.53 ng/mL vs. 772.44±89.36 ng/ml) after the (P<0.001, Figure 4 D).

## 4. Discussion

In the present study, the influence of sex and gonadal hormones on the glutamate concentration after SCI was investigated. The major findings are as follows:

The concentration of baseline glutamate in male rats was significantly greater than female ones; Glutamate level increased after the injury and reached a maximum value at 40 min; Gonadectomy of rats did not significantly change the baseline glutamate levels of the spinal cord in both sexes; and Glutamate concentration increased with the gonadectomy of SCI male animals. In other words, spinothalamic tract axons damage led to a large amount of glutamate release, in this model.
The present results and the data obtained from other models of SCI in previous studies are in agreement, indicating an increase followed by a decrease in glutamate level that finally returns to near baseline level after injury. This shows the same effective mechanisms for secondary damage and glutamate release in this model and other SCI models such as contusion model (Goldshmit et al., 2018; McAdoo & Wu, 2008; Smith et al., 2002; Steward, Popovich, Dietrich, & Kleitman, 2012). Previous studies indicate a correlation between sex differences and central mechanism of feelings, pain processing, and perceptions (Mousavi et al., 2007; Naderi et al., 2014; Taylor, Tio, Paydar, & Sutton, 2018). Several clinical studies indicate the importance of gender in response to neurotrauma (Späni, Braun, & Van Eldik, 2018; Sribnick et al., 2009; Xiong, Mahmood, & Chopp, 2013) and neuroprotective effects of female sex hormones against various aspects of the secondary insults in Cerebrospinal Fluid (CSF) levels of oxidative stress, excitotoxicity markers, and ischemia (Wagner et al., 2007; Zárate, Stevnsner, & Gredilla, 2017).

Large population studies have shown a better functional recovery of women than men following either TBI or SCI (Sribnick, Wingrave, Matzelle, Ray, & Banik, 2003). Although it is difficult to compare the results of SCI and TBI between genders, the epidemiology of these traumatic injuries shows that men are more prone to have these types of injuries than women. Wagner et al. (2007) reported that after severe TBI, the glutamate concentration of male rats CSF was higher than female rats and concluded that gonadal hormones would mediate the gender differences in the function of multiple neurotransmitter systems and brain structure. Testosterone can act directly (by activating androgen receptors) or indirectly on estrogenic pathways (by converting to other steroid hormones) and in both cases, it has a neuroprotective effect (Adhya et al., 2018; Biaek^3^, Zaremba, Borowicz, & Czuczwar, 2004; Woolley & Cohen, 2002).

Generally, there is a positive correlation between the low testosterone in men and chronic SCI (Barbonetti et al., 2014). Thompson and Brenowitz (2010) indicated that local and transsynaptic testosterone could have a neuroprotective effect and prevents the regression of efferent nuclei, respectively. Moreover, testosterone plays a role as a neurotherapeutic agent in the injured nervous system and prevented regressive changes in motoneurons (Byers et al., 2012).

As a neuroprotective agent, testosterone acts through multiple mechanisms, including attenuation of synaptic stripping (Bahnasy, El-Heneedy, & El-Seidy, 2018; Jones, Durica, & Jacob, 1997) and Glial Fibrillary Acidic Protein (GFAP), mediation of the central glial response (Coers, Tanzer, & Jones, 2002; Delchev & Georgieva, 2018; Jones, Coers, Storer, Tanzer, & Kinderman, 1999), and enhancement of the antioxidant enzymes (Ahlbom, Prins, & Ceccatelli, 2001), heat shock protein expression (Mancuso et al., 2018; Tetzlaff, Tanzer, & Jones, 2007; Zhang et al., 2004), ribosomal response (Kinderman & Jones, 1993), and the neurotrophin brain-derived neurotrophic factor (Verhovshek, Cai, Osborne, & Sengelaub, 2010).

Testosterone is also assumed to play a neuroprotective role through the activation of mitogen-activated protein kinase/extracellular signal-regulated kinase signaling pathway (Pike et al., 2008). Catalyzed by the enzyme aromatase (P450aro), estradiol is derived from testosterone (Zheng, 2009). It seems that 17β-estradiol, directly and indirectly, decreases the responses to glutamate activation via the receptor or other mediator levels of the signaling pathway (Weiss et al., 2002). Thus, a neuroprotective activity has been suggested for 17β-estradiol, among neurodegenerative conditions (Platania et al., 2005; Prokai-Tatrai & Prokai, 2018).

Estrogen, as an important gonadal hormone and cerebral protective agent (Rubio, Pérez-Alvarez, Chala, & Wandosell, 2018; Scott, Zhang, Wang, Vadlamudi, & Brann, 2012; Simpkins, Yi, Yang, & Dykens, 2010; Weiss et al., 2002), has a protective effect on neurons (Platania et al., 2005). It prevents neurodegeneration and improves recovery in TBI, SCI, cerebral ischemia and traumatic lesions of the peripheral nervous system (Sribnick et al., 2009; Sribnick et al., 2003; Weiss et al., 2002).

It can also have neuroprotective effects when used before or after an ischemic insult (Weiss et al., 2002). Estrogen inhibits both activation and infiltration of inflammatory cells, attenuates the post-traumatic increase in ion concentration [Ca^2+^] and raises the levels of anti-apoptotic proteins (Fiocchetti, Cipolletti, Ascenzi, & Marino, 2018; Sribnick et al., 2009; Sribnick et al., 2003). It has a protective effect against cell death mediated by glutamate in neurons and neuronal cell lines (Al-Suwailem, Abdi, & El-Ansary, 2018; Hulsebosch et al., 2009; Scott et al., 2012) and prevents Ca^2+^ influx through voltage-gated Ca^2+^ channels. However, it may also act on NMDA receptors that show its potential to restrict secondary cell death due to excitotoxicity (Graham & Scott, 2018; Sribnick et al., 2003).

Estrogen also reduces the response to glutamate receptor activation either by indirect or direct effects on the signal transduction pathway or receptor (Weiss et al., 2002) in order to protect the cells from excitotoxicity. However, the mechanism of such actions of estrogen is unclear (Sribnick et al., 2009). Altogether, it is not known whether the neuroprotective effects of testosterone on glutamate release following SCI are dependent more on the estrogenic pathway or androgenic one.

The results of the present study show that gonadectomy of animals after SCI significantly increased the concentration of glutamate in males but not in female rats. It appears that gonadal hormones in male rats have a greater effect than the female ones on glutamate level after SCI. Altogether, gonadectomy of rats eliminates the main source of testosterone and therefore, eliminates the estradiol as an active metabolite of testosterone. It was also shown that estradiol influences glutamate level. Therefore, the elimination of testosterone in male rats leads to glutamate increase after SCI.

In conclusion, the present data suggest that the concentration of glutamate first increases then decreases, and finally returns to near baseline after the injury. It seems that a low level of testosterone in gonadectomized males may be responsible for the noticeable increase in the spinal cord glutamate concentration after SCI. Therefore, the current results confirm the role of gonadal hormones in the reduction of glutamate level. Further investigations are necessary to clarify the effect of gonadal hormones injection and their receptors antagonist on glutamate concentration after SCI.
